# Supplementary figures and images for: Benchmarking metagenomics classifiers on ancient viral DNA: a simulation study
Source: PeerJ. 2022 Mar 24;10:e12784. doi: 10.7717/peerj.12784 (PMC8958974; doi:10.7717/peerj.12784)

A. Classification per virus

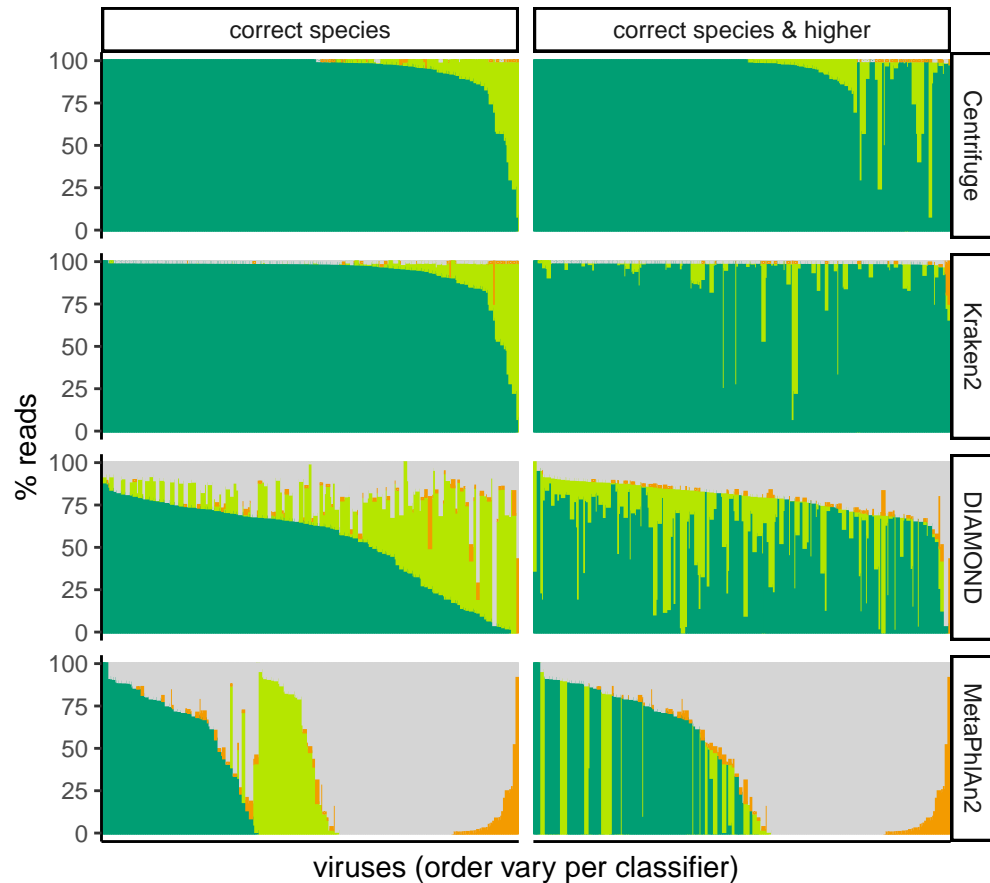

B. Mean classification

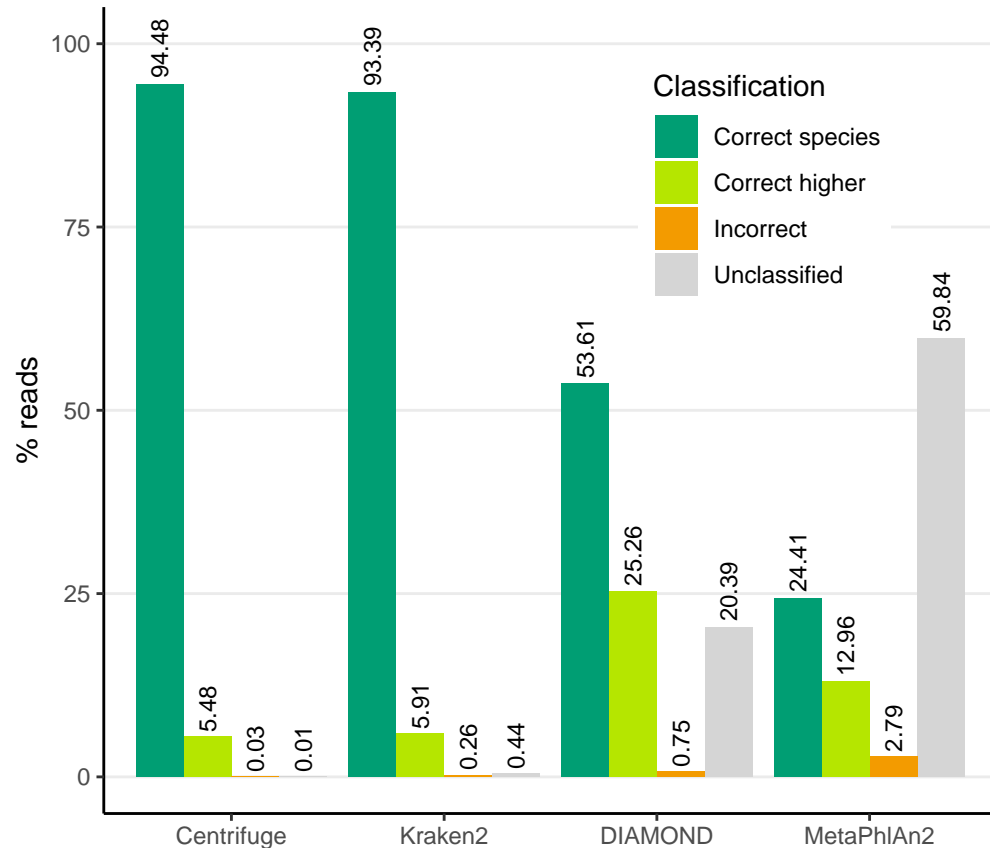

Supplement: Supplemental Information 4 — (A) Percentage of the reads classified in each of the four categories: “correct species”, “correct higher”, “incorrect” and “unclassified” (see Fig. 1) per classifier for each viral sequence. Each bar corresponds to one of the 233 viruses selected for the simulations. Note that the viruses are not ordered the same way for each subplot. (B) Means over the 233 viruses for each classification category. [file peerj-10-12784-s004.pdf]

# Classifier

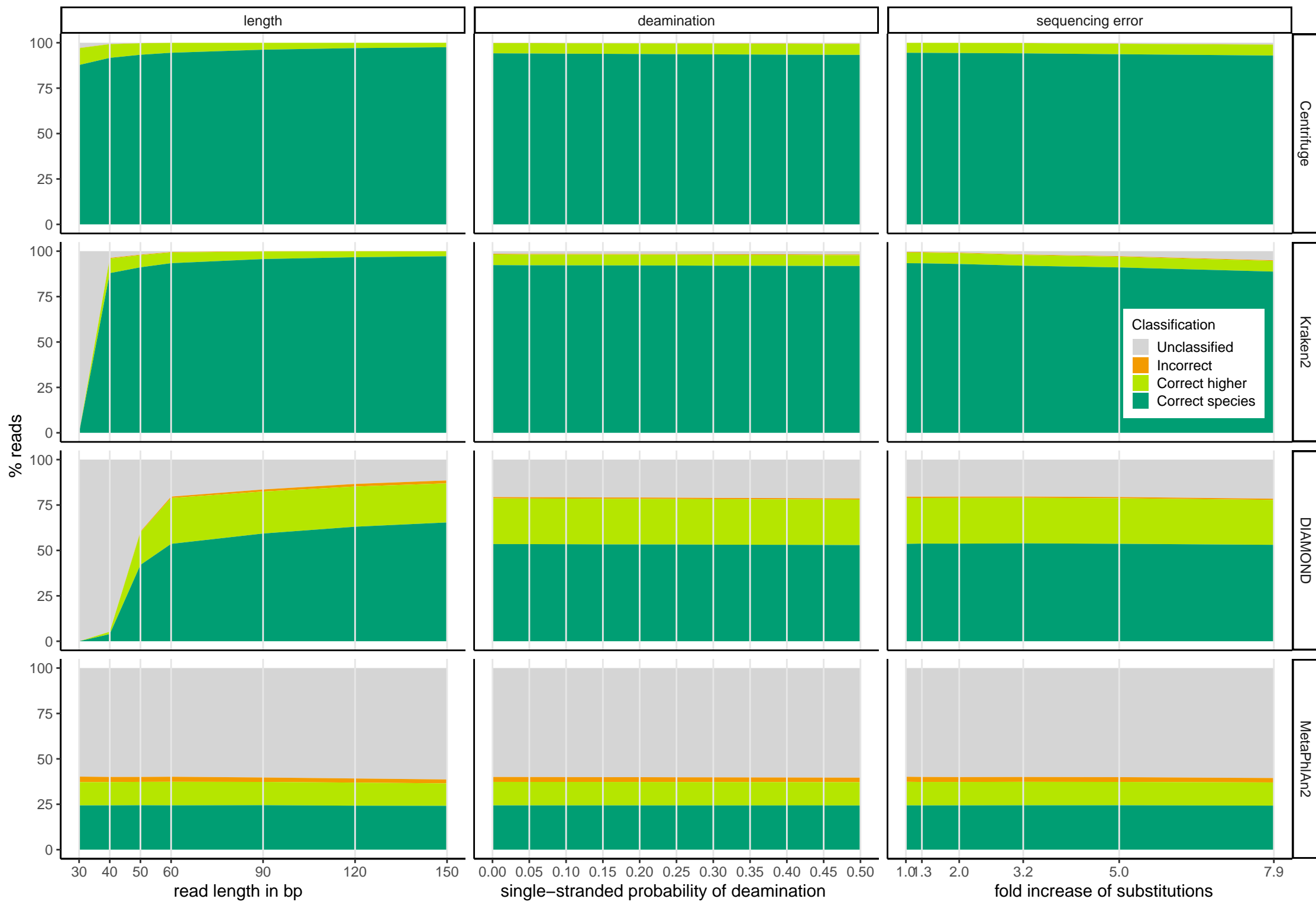

Supplement: Supplemental Information 5 — Nine subplots showing the four classification categories (average across viral sequences) for Centrifuge (first row), Kraken2 (second row), DIAMOND (third row) and MetaPhlAn2 (fourth row) for all simulations; varying read length (first column), singe-stranded probability of deamination (second column), fold increase of substitution sequencing error (third column). [file peerj-10-12784-s005.pdf]

# Classification

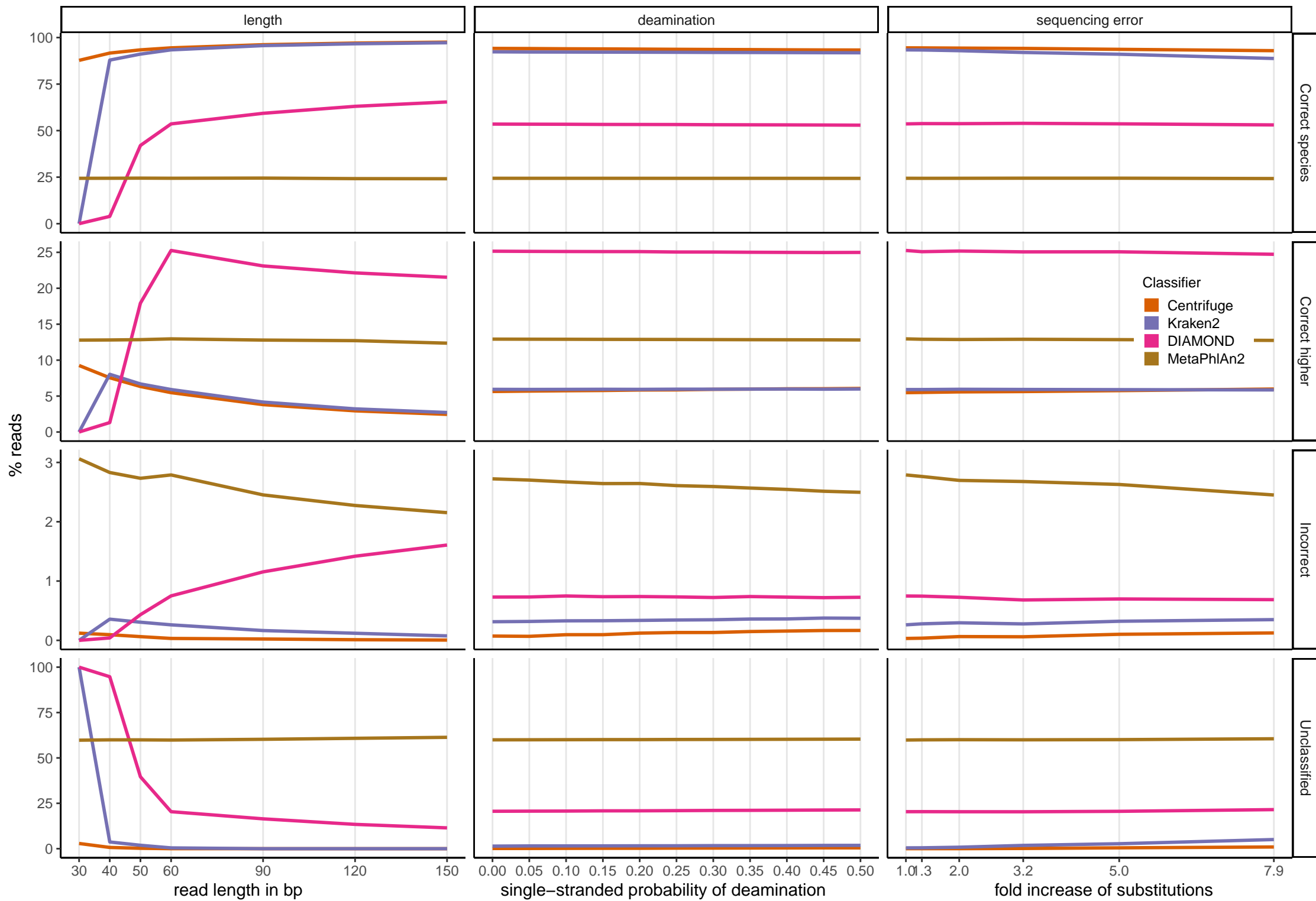

Supplement: Supplemental Information 6 — Twelve subplots showing the four classification categories (average across simulated viral sequences) for the categories “correct species” (first row), “correct higher” (second row), “incorrect” (third row) and “unclassified” (fourth row) for all simulations; varying read length (first column), singe-stranded probability of deamination (second column), fold increase of substitution sequencing error (third column). [file peerj-10-12784-s006.pdf]
