# Supplementary material for: Benchmarking metagenomics classifiers on ancient viral DNA: a simulation study
Source: PeerJ. 2022 Mar 24;10:e12784. doi: 10.7717/peerj.12784 (PMC8958974; doi:10.7717/peerj.12784)
Supplement: Supplemental Information 7 [file peerj-10-12784-s007.docx]

**Supplemental Information 7: MALT**

All the sets of simulated reads mentioned in the main text (with different lengths, deamination and sequencing error values; see Materials and Methods) were also classified with the software MALT (Vågene et al. 2018). In the following supplementary note we present our findings for this classifier.

**Methodology**

***Classification***

MALT version 0.5.3 (Vågene et al. 2018) was used in the nucleotide search mode (-m BlastN) as suggested by (Eisenhofer et al. 2019) for ancient DNA classifications.

***Databases***

As for Kraken2, Centrifuge and DIAMOND (see Supplementary Note 2), we tried to build two databases for MALT: a “full” database including RefSeq sequences for archaea, bacteria, viruses and human; and a viral database only including all the viral sequences from RefSeq. However, the creation of the full database required more than 1 TB of memory, which exceeds our resources. As a result, we only built the viral database. We presume that this extensive memory usage will also cause a problem to other researchers, limiting the usage of exhaustive MALT databases.

***Reference sequences***

To build the viral database we used all viral sequences from RefSeq, namely viral.1.1.genomic.fna.gz, viral.2.1.genomic.fna.gz and viral.3.1.genomic.fna.gz (downloaded from ftp.ncbi.nlm.nih.gov/refseq/release/viral/ on 27 June 2021).

***Accession to taxonomy translation***

Following the manual (https://software-ab.informatik.uni-tuebingen.de/download/malt/manual.pdf), we searched for the accession to taxon translation file within the installation folder of MALT, but there was no such file. We therefore downloaded the accession to taxon translation file *nucl_wgs.accession2taxid.gz* from NCBI (downloaded from ftp.ncbi.nlm.nih.gov/pub/taxonomy/accession2taxid/ on 27 June 2021) and built the database with the following command:

malt-build -i foo.fna -s DNA --index dbName --acc2taxa nucl_wgs.accession2taxid.gz

While the database creation worked well (in the sense that there were no errors reported) and while the overall results looked reasonable (see Figure SN1-1, NCBI-acc2taxa), inconsistent results were observed. In summary (but see also below), in some cases, the alignment done by MALT is correct but the reported taxon is wrong.

We assumed that there was an issue within the accession number to taxon ID translation. We therefore exchanged the translation file to megan-nucl-Jan2021.db.zip, a translation file provided by the authors available on their website for MEGAN, a related software (https://software-ab.informatik.uni-tuebingen.de/download/megan6/welcome.html; file downloaded on 17 February 2021).

Again, the database creation worked well (there was no error or warning reported). The classification performance was also similar to the previous classifications (see Figure SN1-1, MEGAN-acc2taxa below). But again, we detected incorrect translations of the sequence accession number to the taxonomic ID (taxID).

Using the command line manual (“malt-build --help”), we realized that (even though the software ran without errors and warnings) the command line manual and MALT’s pdf manual are not in line with each other and that the parameter “acc2taxa” we used to pass the translation file was likely deprecated. We repeated the analyses following the command line manual and used then the parameter “mapDB” to pass the translation file:

malt-build -i foo.fna -s DNA --index dbName --mapDB megan-nucl-Jan201.db

With this new database the classification performance (sensitivity and precision) increased substantially (Figure SN1-1, MEGAN-mapDB) and we chose to analyse those results in more details (see below). However, issues with the undetected viruses persisted presumably also due to accession to translation file.

**Results**

**Classifications for 60 bp simulations**

The 60 bp simulations results for three combinations of translation files and parameters mentioned in the methodology above are shown hereafter.

Figure SN1-1. A) Percentage of the reads classified in each of the four categories: “correct species”, “correct higher”, “incorrect” and “unclassified” (see Figure 1) per “translation file” for MALT. Each bar corresponds to one of the 233 viruses selected for the simulations. B) Means over the 233 viruses for each classification category. “NCBI-acc2taxa” corresponds to the run with nucl_wgs.accession2taxid.gz used as translation file; “MEGAN-acc2taxa” to the run with megan-nucl-Jan2021.db.zip used as translation file; and “MEGAN-mapDB” to the run with megan-nucl-Jan2021.db.zip used as translation file and the parameter mapDB to pass translation.

As can be seen in Figure SN1-1 A, most viruses are recovered and the number of correctly classified reads is quite high per virus, suggesting the software was run successfully. However, when we analysed further the viruses that were not identified by MALT (see last section), we detected incorrect translations of the sequence accession number to the taxonomic ID (taxID). More specifically, there are instances where the alignment done by MALT is correct, but the reported taxon is wrong. This includes, surprisingly, classifications with reported taxa not present in the database. Note that although this issue is common to all our attempts, we compare MALT to the other four classifiers presented below for our best run, i.e. the case with the MEGAN translation file (megan-nucl-Jan201.db) and with the --mapDB parameter (“MEGAN-mapDB” in Figure SN1-1 A).

**MEGAN accession to taxonomy translation file with command mapDB**

For the 60 bp simulations, MALT shows sensitivity and precision values close to those for Centrifuge and Kraken2 and which outperform DIAMOND and MetaPhlAn2 (Figure SN1-2 A), both when considering correct species only and when including the higher taxa, with one exception: DIAMOND has a higher precision value when taking into account the higher taxa. A total of 221 viruses out of the 223 are identified correctly with MALT (less than Centrifuge, Kraken2 and DIAMOND; more than MetaPhlAn2) (Figure SN1-2 B). Among all the classifiers MALT reports the highest number of spurious extra taxa (Figure SN1-2 C).

Figure SN1-2. A) Mean sensitivity versus mean precision. The mean sensitivities (Sensitivity_s & Sensitivity_s&h) are the means of the proportions of reads correctly classified over the total number of simulated reads across viruses. The mean precisions (Precision_s & Precision_s&h) are the means of the proportions of reads correctly classified over the number of classified reads across viruses. Circles denote the values if only “correct species” reads are considered as correctly classified reads; triangles denote the values if “correct species” and “correct higher” reads are considered as correctly classified reads (see Materials and Methods). The perfect classifier would have 100% sensitivity and 100% precision. B) Total number of viruses recovered for each classifier when correctly identifying at least 1 read per virus. The dashed line indicates the total number of tested viruses (233). C) Mean number of spurious extra taxa per classifier. In this plot, a taxon is assumed as identified by a classifier if at least 1 read is assigned to it.

When varying the read length MALT ranks mostly as the third classifier with the highest values of sensitivity and precision (Figure SN1-3 A & B). At 30 bp MALT outperforms Kraken2, and even Centrifuge when considering only species level; although Centrifuge remains the classifier with the best performance for the rest of the lengths (Figure SN1-3 A & B). DIAMOND outperforms MALT in precision when higher taxa are included (Figure SN1-3 B). The number of identified viruses for MALT is the same across all the tested lengths (Figure SN1-3 C). For reads of length 30 bp and 40 bp MetaPhlAn2 reports the highest number of spurious extra taxa; MALT has the highest number for 50 bp, 60 bp and 150 bp; while DIAMOND has the highest number for 120 bp (Figure SN1-3 D).

Figure SN1-3. For these simulations, read length was varied from 30 to 150 bp. A) Average Sensitivity_s (continuous lines) and Sensitivity_s&h (dashed lines) for each classifier. B) Average Precision_s (continuous lines) and Precision_s&h (dashed lines) for each classifier. C) Total number of viruses detected out of the 233 tested. The dashed line shows the maximum number of detectable viruses. D) Average number of spurious extra taxa across simulated viral sequences. The vertical dashed line indicates the initial 60 bp read set.

By adding deamination damage MALT has a slightly higher sensitivity (+~1%), only species level considered than Kraken2 (Figure SN1-4 A). However, if the correct higher taxa are considered, Kraken2 outperforms MALT both in sensitivity and precision (Figure SN1-4 B). Once more, DIAMOND has higher precision values than MALT by considering the correct high taxonomic classifications (Figure SN1-4 B); Centrifuge has the highest sensitivity and precision (Figure SN1-4 A & B); the number of identified viruses by MALT remains unaltered (Figure SN1-4 C); and MALT presents the highest number of spurious extra taxa (Figure SN1-4 D).

Figure SN1-4. For these simulations, errors were added using deamSim gargamel subprogram which assumes an ancient DNA deamination-like distribution and were added in addition to the ART Illumina like sequencing errors. The results shown here correspond to a single-stranded probability of deamination varying from 0 to 0.5. For all the results the nick frequency is set at 0.03, the average length of overhanging ends is set at 0.25, and the probability of deamination in the double-stranded portions of DNA is set at 0.01. A) Average Sensitivity_s (continuous lines) and Sensitivity_s&h (dashed lines) for each classifier. B) Average Precision_s (continuous lines) and Precision_s&h (dashed lines) for each classifier. C) Total number of viruses detected out of the 233 tested. The dashed line shows the maximum number of detectable viruses. D) Average number of spurious extra taxa across simulated viruses.

In the sequencing error simulations we observed that MALT has higher sensitivity (only considering species level) than Kraken2 for the lower qShift values (–5, –7 and –9, i.e. higher sequencing error) (Figure SN1-5 A). As with the deamination simulations Centrifuge outperforms all the tools (Figure SN1-5 A & B); DIAMOND has a higher precision (adding higher taxa) than MALT (Figure SN1-5 B); the number of identified viruses by MALT is the same across all qShift values (Figure SN1-5 C); and MALT remains as the tool with the highest number of spurious extra taxa (Figure SN1-5 D).

Figure SN1-5. For these simulations, errors were added using ART which assumes a profile similar to the ones observed for Illumina Sequencing machines (HiSeq 2500). The results shown here correspond to increasing the overall sequencing error rate ranging from 1 to 7.9-fold (qShift values from 0 to -9). On the x-axis, the first number correspond to the expected fold increase in error rate while the parameter that was varied, *qShift*, is shown in parenthesis. A) Average Sensitivity_s (continuous lines) and Sensitivity_s&h (dashed lines) for each classifier. B) Average Precision_s (continuous lines) and Precision_s&h (dashed lines) for each classifier. C) Total number of viruses detected out of the 233 tested. The dashed line shows the maximum number of detectable viruses. D) Average number of spurious extra taxa across simulated viral sequences. The vertical dashed line indicates the initial 60 bp read set.

***Unidentified viruses and unexpected results***

Finally, we analysed further the viruses that were not identified by MALT. By doing so, we detected incorrect translations of the sequence accession number to the taxonomic ID (taxID). Despite using different translation files (see above) and having changed the way we run the software, we still found discrepancies between MALT’s alignments and MALT’s taxonomic assignments. We found examples of three types of issues: 1) the read aligned to a specific taxon but is assigned incorrectly to a different one, 2) classifications that could be correct (the read aligned correctly to the virus tested, but did not have a taxonomic assignment), as well as, 3) classifications that could be incorrect (the read aligned incorrectly to another species rather than to the virus tested, but it did not have a taxonomic assignment). In the first case, and more specifically, it happens that MALT’s alignment is to a specific taxon, but that the related reported taxon is a different one. This can lead to incorrect classifications with reported taxon not present in the database. For instance, the virus human papillomoavirus type 5 is aligning to Colobus guereza papillomavirus type 2, but the classification reports the primate *Colobus guereza,* a taxon not present in the database used. As an example of the second case, we identified reads generated from IAS virus which were correctly aligned only to the IAS virus, but MALT did not report any reads assigned to this taxon. Finally, as an example of the third case, within the same set of IAS virus simulated reads, there were many reads uniquely aligned to crAssphage cr116_1, crAssphage cr128_1, crAssphage cr85_1 and to other members of the crAss-like viruses clade (all of them are taxa outside of the tested virus tree, i.e. they would be incorrect classifications), but none of these taxa are reported by MALT.

Note that some of the same issues has been already reported in 2018 (<http://megan.informatik.uni-tuebingen.de/t/megan-assignation-after-malt/753/2>) but there does not seem to be an answer. To try to resolve the issue we looked into the code, contacted the authors (<http://megan.informatik.uni-tuebingen.de/t/malt-reports-classifications-outside-of-db-range/1909>) and asked the “SPAAM” community (https://spaam-community.github.io/). We have not been able to resolve the issue so far.

To conclude, even though MALT looks very promising, given these results we cannot explain, it is unclear to us if the overall classification results we obtained are trustworthy.
