# Supplementary material for: Benchmarking metagenomics classifiers on ancient viral DNA: a simulation study
Source: PeerJ. 2022 Mar 24;10:e12784. doi: 10.7717/peerj.12784 (PMC8958974; doi:10.7717/peerj.12784)
Supplement: Supplemental Information 8 [file peerj-10-12784-s008.docx]

**Supplemental Information 8: archaeal, bacterial, human and viral databases for Centrifuge, Kraken2 and DIAMOND**

In this section we present the classification results of the read length simulations (see Materials and methods) obtained with Kraken2 and Centrifuge’s default databases (i.e. the databases that include archaeal, bacterial, human and viral data), as well as with an archaeal, bacterial, human and viral database for DIAMOND. Note that the results presented in the main text were obtained with viral databases for all classifiers except MetaPhlAn2.

**Methodology**

We built Centrifuge and Kraken2’s databases following the developers’ instructions. Centrifuge database includes RefSeq sequences from archaea, bacteria, human and viruses (built on 2 September 2021); Kraken2 database in addition has sequences from UniVec_Core (downloaded 17 May 2021). To build DIAMOND’s database we used protein sequences from archaea, bacteria, human and viruses (so that all databases were comparable), downloaded from RefSeq on 6 September 2021. In the following lines we will refer to these databases as “full databases”.

**Results**

In the initial 60 bp reads simulation we observed a better performance of the classifiers using the viral database (Figure SN2-1 & Figure SN2-2). The number of correct species and correct higher classification is greater when using viral databases for all three classifiers. In addition, the number of incorrect classifications and unclassified reads are reduced for the viral databases (compared to the full databases). This is likely explained by the greater number of species in the full databases, many of them can confound the taxonomic assignments as they could resemble the tested viruses (Figure SN2-1).

In the specific case of DIAMOND, the proportion of unclassified reads reaches 67.5% when using the full database. As the full database stores a greater number of sequences than the viral database the e-values of DIAMOND alignments increases; it therefore seems that many reads do not pass DIAMOND’s threshold resulting in a greater proportion of unclassified viruses.

Figure SN2-1. Mean percentage over the 233 human DNA viruses of reads classified in each of the four categories: “correct species”, “correct higher”, “incorrect” and “unclassified” (see Figure 1). The bars with the suffix “_viral” show the means obtained with the “viral” databases. The bars with the suffix “_full” show the means obtained with the “full” databases, which contain archaeal, bacterial, human and viral sequences.

The aforementioned proportions are reflected in the mean sensitivity and precision values: the viral databases sensitivity and precision scores are higher than the full databases ones, both when considering only species level and when including higher taxa (Figure SN2-2 A & B). This difference is accentuated in the sensitivity, given that this measure considers all reads and the proportion of unclassified reads is higher with the full databases. The exception are Kraken2 and DIAMOND, which have slightly higher values for precision_s when using its full database (Figure SN2-2 B).

Regarding the number of identified viruses: Kraken2 and Centrifuge identify more viruses with the viral database, while DIAMOND detects the same number of viruses regardless the database used (Figure SN2-2 C). When checking Kraken2 and Centrifuge databases we found out that their default databases (full databases) did not include sequences for 1 virus in the case of Kraken2, and for 6 in the case of Centrifuge. We tried to re-build these databases several times but the viruses were missing, we do not know why the sequences are not automatically downloaded for those species during the steps to build a database for those two tools.

As discussed above, for all the classifiers we observed a higher amount of spurious extra taxa reported when they use their full databases compared to the viral database. This is expected as there are more possible confounding species in the full databases (Figure SN2-2 D).

Figure SN2-2. A) Mean sensitivity over the 233 human DNA viruses. B) Mean precision over the 233 human DNA viruses. Darker grey bars show sensitivity and precision when considering species level only, light grey bars show sensitivity and precision when considering species level and higher taxonomic ranks together C) Total number of correctly detected viruses over the 233 tested. D) Mean number of reported spurious extra taxa over the 233 human DNA viruses. The bars with the suffix “_viral” show the results obtained with the “viral” databases. The bars with the suffix “_full” show the results obtained with the “full” databases, which contain archaeal, bacterial, human and viral sequences.

Similar trends to the 60 bp reads simulation are observed when varying the read length: higher sensitivity and precision with the viral databases; and a higher amount of spurious extra taxa with full databases (Figure SN2-3).

Figure SN2-3. Comparison of “viral” databases (darker colors) vs “full” databases (lighter colors) output. Simulated reads of different lengths (x axis) from the 233 viruses were used (see Materials & Methods in main). A) Average Sensitivity_s (continuous lines) and Sensitivity_s&h (dashed lines) for each classifier and for each database. B) Average Precision_s (continuous lines) and Precision_s&h (dashed lines) for each classifier and for each database. C) Total number of viruses detected out of the 233 tested. The dashed line shows the maximum number of detectable viruses. D) Average number of spurious extra taxa across simulated viral sequences. The vertical dashed line indicates the initial 60 bp read set.

**Discussion**

In summary, as the goal with the classification step in an ancient DNA study is to increase the number of true candidates that can be further explore, we believe that the viral database is the most appropriate choice of database to detect ancient (human) DNA viruses. We have made those databases available.
