# Supplementary material for: Benchmarking metagenomics classifiers on ancient viral DNA: a simulation study
Source: PeerJ. 2022 Mar 24;10:e12784. doi: 10.7717/peerj.12784 (PMC8958974; doi:10.7717/peerj.12784)
Supplement: Supplemental Information 9 [file peerj-10-12784-s009.docx]

**Supplemental Information 9: New parameters for DIAMOND and Kraken2 to handle short reads**

As discussed in the main text, with default parameters, short reads cannot be “handled” by DIAMOND and Kraken2 (see Figure 5 in main, there are no classifications for neither of them). In this supplementary note, we implemented a set of simulations to test the performance of DIAMOND and Kraken2 with parameters that allow both of them to classify short reads (30 bp long).

**Methodology**

**DIAMOND:**

For this set of simulations, we reduced the length of DIAMOND’s seed to 9 aminoacids with the parameter *--shape-mask 111111111*, which can align reads of length 27 bp or longer. The rest of the parameters and the database (viral sequences database) remained the same (default parameters).

**Kraken2:**

In the case of Kraken2 we built a new database (with the same sequences as the default one: archaeal, bacterial, human, viral and UniVec_Core sequences) with k-mers of length 29 using the parameter *--kmer-len 29* (i.e. in theory, reads of 29 bp or longer can be classified). The rest of the parameters and the database (viral sequences database) remained the same.

**Results**

With these new simulations, the reads were classified for different read lengths (30, 40, 50, 60, 90, 120 and 150 bp long) generated out of the 233 viruses (see main Materials and methods, Simulation of viral reads and read length); and the outputs of DIAMOND and Kraken2 were compared for default parameters versus a seed of 9 amino acids (DIAMOND) and 29 bp-kmer (Kraken2). The main results are shown below:

Figure SN3-1. DIAMOND and Kraken2’s parameters for short reads. DIAMOND (pink colors) and Kraken2 (purple colors) comparison of default parameters (darker colors) versus parameters tailored for short reads (lighter colors). Simulated reads of different lengths (x axis) from the 233 viruses were used (see Materials & Methods in main). A) Average Sensitivity_s (continuous lines) and Sensitivity_s&h (dashed lines) for each classifier and for its different parameters. B) Average Precision_s (continuous lines) and Precision_s&h (dashed lines) for each classifier and for its different parameters. C) Total number of viruses detected out of the 233 tested. The horizontal dashed line shows the maximum number of detectable viruses. D) Average number of spurious extra taxa across simulated viral sequences. The vertical dashed line indicates the initial 60 bp read set.

By changing the parameters used to build the database (Kraken2) or to run the software (DIAMOND), both Kraken2 and DIAMOND are able to classify 30 bp reads (Figure SN3-1). Kraken2 with 29 bp k-mers has almost a sensitivity of 50% which contrasts with the 0% sensitivity of Kraken2 with 35 bp k-mers (default k-mer length), however as the read length increases, the sensitivity of Kraken2 with 35 bp k-mers and the sensitivity of Kraken2 with 29 bp k-mers reach a similar value (Figure SN3-1 A). Something similar happens with Kraken2’s precision, although the precision is less affected (Figure SN3-1 B). Regarding the number of detected viruses both k-mers identify the same number across all lengths. Remarkably, Kraken2 with 29 bp k-mers also identifies the same viruses in the 30 bp reads simulations (Figure SN3-1 C). However, a considerable disadvantage observed when using 29 bp k-mers is the increase of spurious taxa reported by Kraken2; across all lengths the mean number of spurious extra taxa is considerably larger when using 29 bp k-mers compared to the default 35 bp-kmers (Figure SN3-1 D). In DIAMOND’s case, the number of reads recovered in the 30 bp simulations with a shorter seed of 9 aminoacids is minimal (Figure SN3-1 A). In all cases tested (sensitivity, precision, detected viruses and mean number of spurious taxa) there is almost no difference of using a shorter seed of 9 aminoacids or using DIAMOND’s default seeds (Figure SN3-1).

**Discussion**

Considering these mixed results, we present the default parameters in the main text. Considerably more work is needed to find out the best combination of parameters for those tools for ancient DNA short reads.
