# Supplementary material for: Benchmarking metagenomics classifiers on ancient viral DNA: a simulation study
Source: PeerJ. 2022 Mar 24;10:e12784. doi: 10.7717/peerj.12784 (PMC8958974; doi:10.7717/peerj.12784)
Supplement: Supplemental Information 10 [file peerj-10-12784-s010.docx]

**Supplemental Information 10: Unclassified viruses and viruses with high incorrect classifications**

**Unclassified viruses**

None of the classifiers identified all the 233 viruses tested. The failure of identifying some viruses can be explained mainly by two reasons: either the virus sequence is very similar to sister species in the database or the virus was not represented on the database. In the following lines we give a detailed explanation of each of the missing viruses by each of the classifiers tested.

**Kraken2**

Kraken2 failed to identify human adenovirus 2 (AC_000007.1) because there is no minimizer specific to this serotype in the database, then the virus is mostly classified at a higher level (species) to human mastadenovirus C (5,943 reads). There were as well a couple of reads correctly classified at the genus level and few reads (6) incorrectly classified that were confounded with sister serotypes (human adenovirus 1 and human adenovirus 5).

**DIAMOND**

Within the 5 viruses not identified by DIAMOND, 3 of them have no proteins in RefSeq: porcine endogenous retrovirus E (NC_003059.1), senegalvirus marseillevirus (NC_04322[3-8]).1 and torque teno virus (NC_015783.1). Porcine endogenous retrovirus E and senegalvirus marseillevirus have correct classifications at higher taxonomic levels; while torque teno virus only have reads incorrectly classified given that most of the alignments were to species in the genus *Alphatorquevirus*, whereas torque teno virus is catalogued within the unclassified *Anelloviridae* rank. The other two viruses that DIAMOND was not able to identify were human adenovirus 2 (AC_000007.1) and human polyomavirus 12 (NC_020890.1), both of them had alignments but also to close sister species, then DIAMOND classified their reads as “correct higher” instead of “correct species”.

**Centrifuge**

As Kraken2 and DIAMOND, Centrifuge failed to identify human adenovirus 2 (AC_000007.1). As Centrifuge compresses genomes from strains of the same species (Kim et al. 2016) almost all alignments were against the species level human mastadenovirus C, just two reads were misclassified as the sister serotype human adenovirus 1.

**MetaPhlAn2**

Finally, in MetaPhlAn2’s case 146 viruses do not have any “correct species” reads. Out of the 151 viral sequences with no reads classified correctly at species level, 65 of them do not have any classifications, 33 have classifications only at a higher taxonomic level, 37 have only incorrect classifications and 16 have both high correct and incorrect classifications. Out of the 146 viruses, only one has a marker gene associated to its accession number in MetaPhlAn2’s database: porcine endogenous retrovirus E (NC_003059.1), which is correctly classified but at a higher taxonomic level.

**Incorrect classifications**

Some of the tested viruses present high proportions of incorrectly classified reads for specific classifiers. As with the undetected viruses we observed that these misclassifications are due to the absence of the virus in the database, or because there are many sister species that confound the taxonomic assignment. Encouragingly, for almost all viruses across all classifiers, the incorrect proportion does not exceed 40%. In the following lines we discuss which were the viruses with the highest proportions of incorrect classifications per classifier.

**Kraken2**

Kraken2 has only four viruses with a proportion of incorrectly classified reads higher than 1%. adeno-associated virus - 8 (NC_006261.1) is the virus with the highest incorrect proportion with 24.5% incorrectly assigned reads, all of them assigned to close sister species; followed by adeno-associated virus - 7 (NC_006260.1) with 18.2%, adeno-associated virus - 3 (NC_001729.1) with 8.4% and adeno-associated virus - 4 (NC_001829.1) with 6.4%, in all the cases the incorrect assignments were to sister species within the adeno-associated dependoparvovirus A rank.

**Centrifuge**

For Centrifuge all the tested virus has less than 1% incorrect classified reads. The virus with the highest proportion of incorrect reads was cyclovirus VN (NC_039215.1) with 0.33%, the incorrect assignment was to the sister species cyclovirus TN25.

**DIAMOND**

In the case of DIAMOND, torque teno virus (NC_015783.1) has the highest incorrect proportion of classified reads with 42.7% as this virus has no proteins stored in RefSeq; all classified reads were incorrectly assigned to species in the genus *Alphatorquevirus*, whereas torque teno virus is catalogued within the unclassified *Anelloviridae* rank. The virus with the second highest incorrect proportion is gemycirculavirus C1c (NC_038496.1) with 29.7%, the incorrect assignments were to mongoose feces-associated gemycircularvirus c and porcine feces-associated gemycircularvirus, both of them belong to the same genus (*Gemykibivirus*) as the tested virus. The contigs from senegalvirus marseillevirus (NC_04322[3-8].1) also present high proportions of incorrect assignments ranging from 26.1% to 7.6%. As with torque teno virus this virus does not have any protein in RefSeq, the incorrect assignments are within the *Marseillevirus* genus.

**MetaPhlAn2**

In the case of MetaPhlAn2 the viruses with the highest proportions of incorrectly classified reads are not present in its database. Human papillomavirus 179 (NC_022095.1) has an incorrect proportion of 91.3%, with all reads classified as human papillomavirus which belongs to the clade human papillomavirus types, while human papillomavirus 179 belongs to the *Gammapapillomavirus* genus. It is followed then by human parvovirus 4 G1 (NC_007018.1) with 89%, all reads were classified as the sister species human parvovirus 4. Then we have human bocavirus 2c PK (NC_012042.1) with 51.6%, all the assignments were to the sister species human bocavirus 2 and to bocavirus gorilla/GBoV1/2009, a member of the same genus (*Bocaparvovirus*) as human bocavirus 2c PK.
